# Supplementary material for: Stable and bright formamidinium-based perovskite light-emitting diodes with high energy conversion efficiency
Source: Nat Commun. 2019 Aug 9;10:3624. doi: 10.1038/s41467-019-11567-1 (PMC6689020; doi:10.1038/s41467-019-11567-1)
Supplement: Supplementary file 1 — Supplementary Information [file 41467_2019_11567_MOESM1_ESM.pdf]

Supplementary Information

Stable and bright formamidinium-based perovskite light-emitting diodes with high energy conversion efficiency

Miao *et al.*

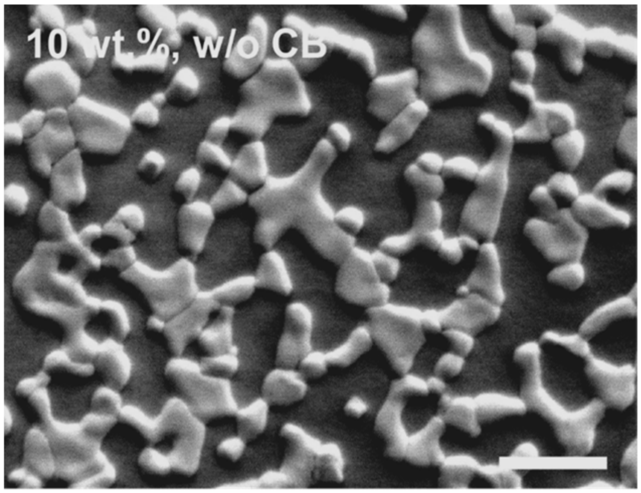

Supplementary Figure 1. SEM image of 10 wt.% $FAPbI_3$ film fabricated without CB as anti-solvent. Scale bar, 1 μm.

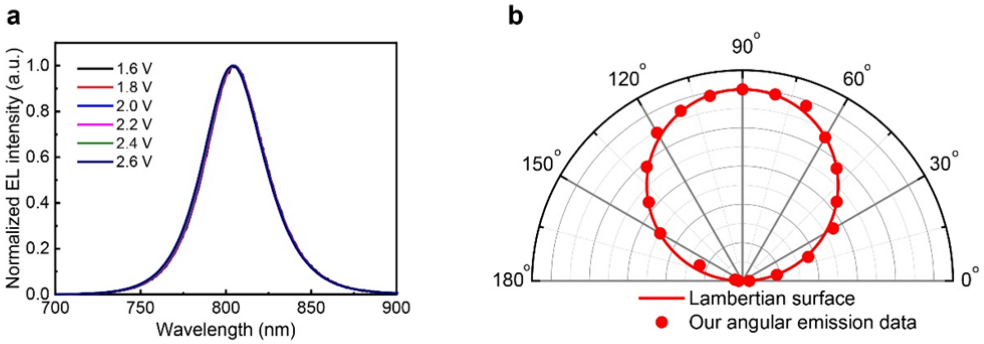

Supplementary Figure 2. a, Normalized device EL spectra upon various biases. **b,** Angular distribution of radiation intensity for $FAPbI_3$ LEDs.

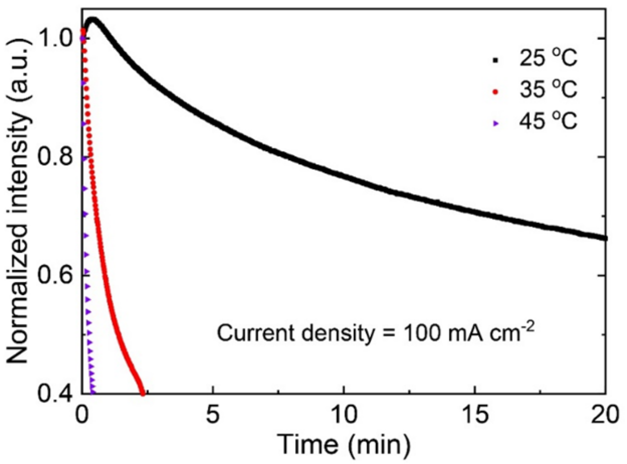

Supplementary Figure 3. Temperature dependent EL decay of 10 wt.% PeLEDs. Devices were tested in the glovebox. The result shows that the stability is strongly dependent on the temperature.

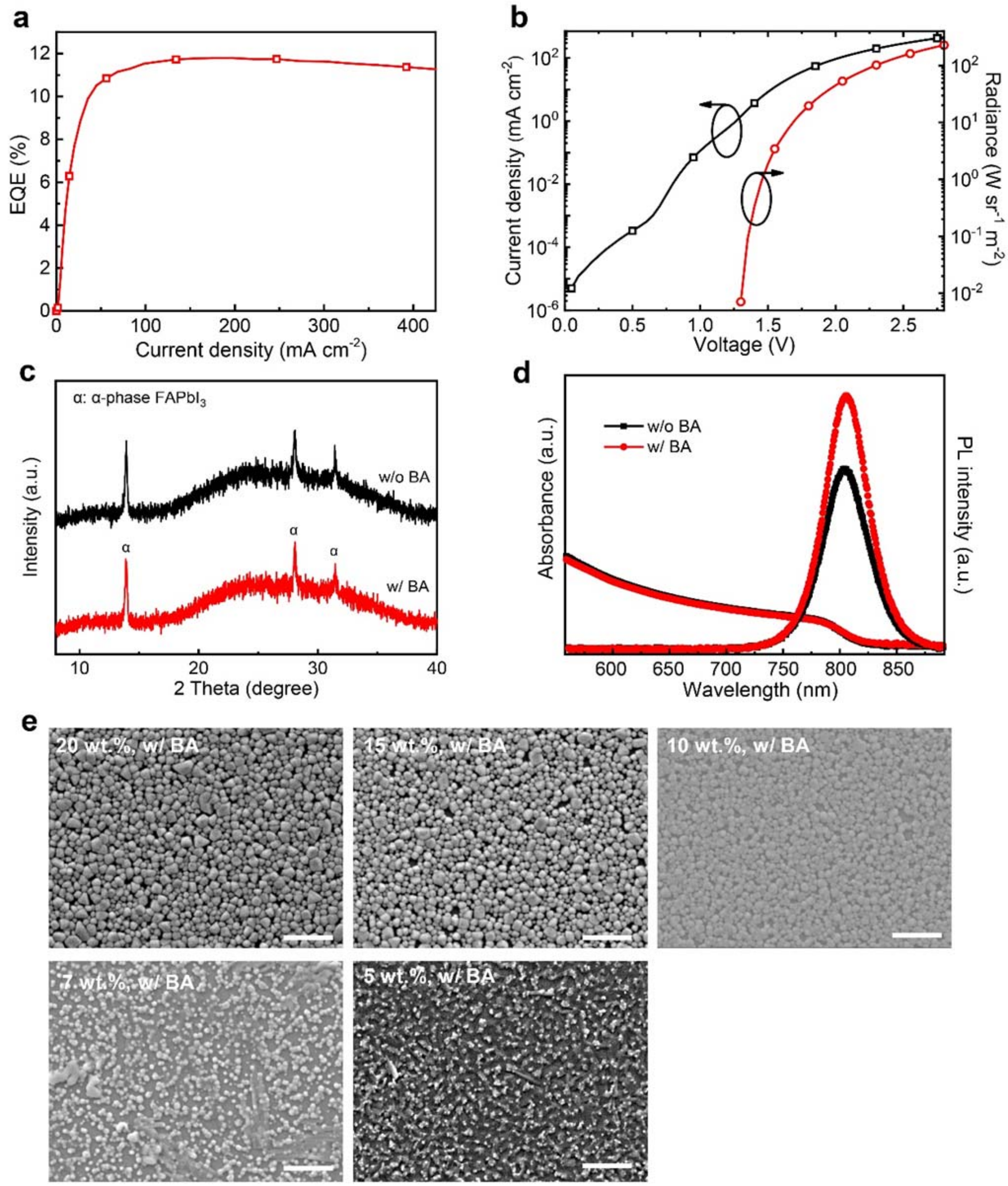

Supplementary Figure 4. Characteristics of the BA treated PeLEDs and films. a, EQE versus current density of the BA treated PeLED. A peak EQE of 11.8% is achieved under a current density of 175 mA cm^{-2}. **b**, Dependence of current density and radiance on the driving voltage. A radiance of 241 W $sr^{-1}m^{-2}$ is obtained under 2.85 V. **c,** XRD patterns of the 10 wt.% $FAPbI_3$ films with and without BA treatment. The XRD peak is almost identical, and no peaks from layered perovskite observed. **d,** Absorption and PL spectra of the 10 wt.% $FAPbI_3$ films with and without BA

treatment. **e,** SEM images of $FAPbI_3$ films fabricated with different concentrations of precursor solutions with BA treatment. Scale bar, 1 μm.

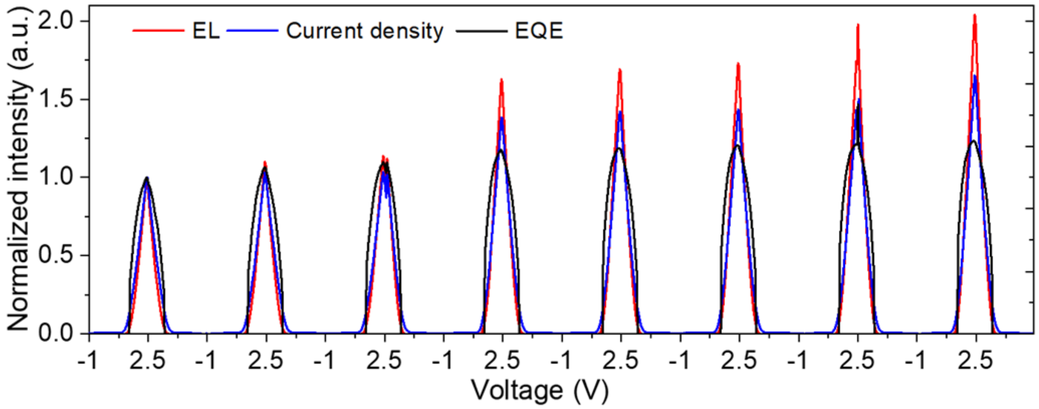

Supplementary Figure 5. EL/current density/EQE under cyclic bias between -1 to 2.5 V, normalized to individual initial data of the first cycle.

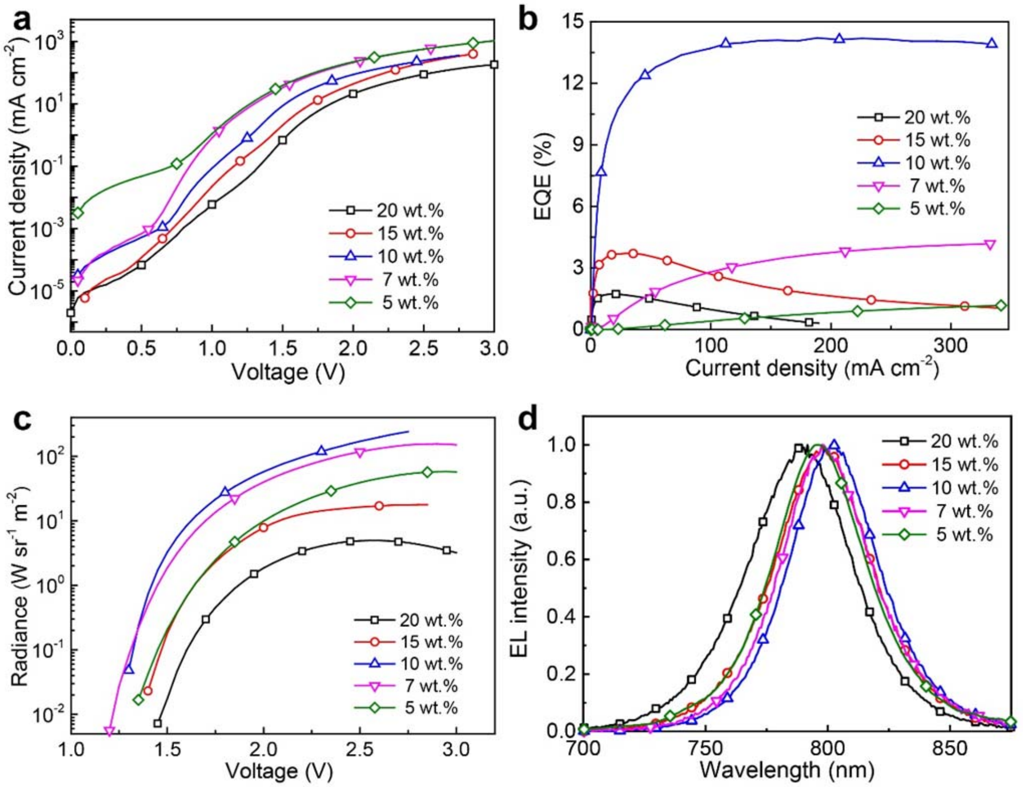

Supplementary Figure 6. Optoelectronic characteristics of PeLEDs. The devices were fabricated with different precursor concentrations. **a,** Current density versus driving voltage. **b,** EQE versus current density. **c,** Radiance versus driving voltage. **d,** EL spectra under a driving voltage of 2.45 V. The EL peaks for 20 wt.%, 15 wt.%, 7 wt.% and 5 wt.% perovskite films are 790, 798, 798 and 796 nm respectively.

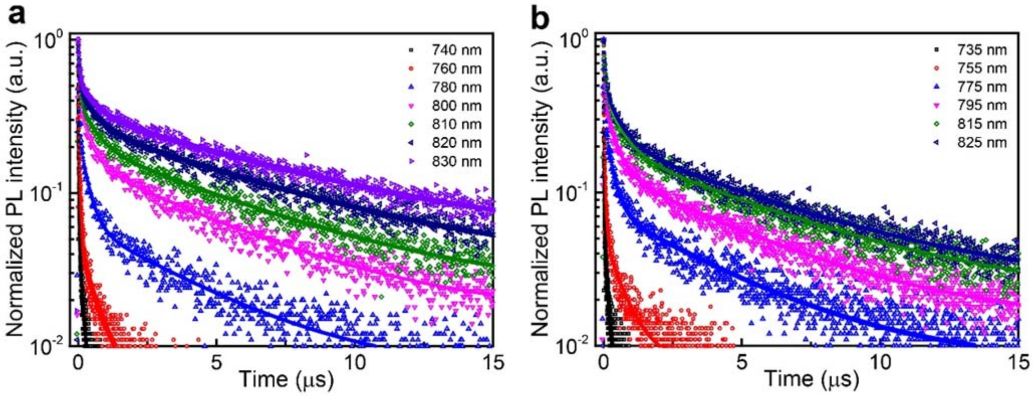

Supplementary Figure 7. Time-resolved PL of perovskite films at various emission wavelengths. The films are fabricated with different concentrations of precursor solutions. **a,** 20 wt.%. **b,** 15 wt.%.

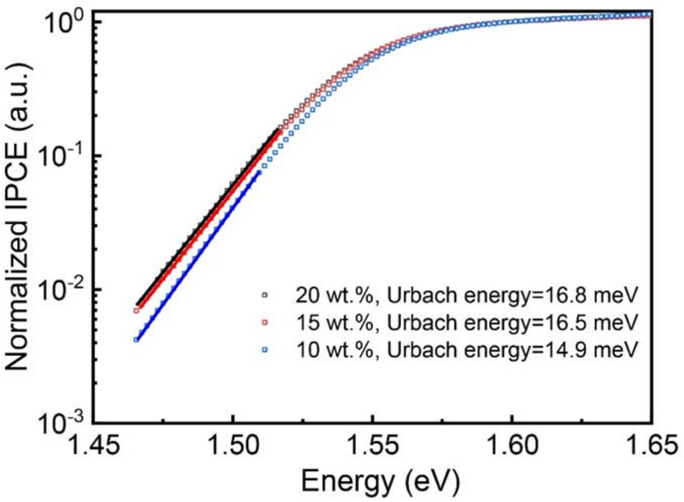

Supplementary Figure 8. Normalized IPCE at the absorption onset. PeLEDs are fabricated with 20 wt.%, 15 wt.%, and 10 wt.% $FAPbI_3$ films, measured by using FTPS.

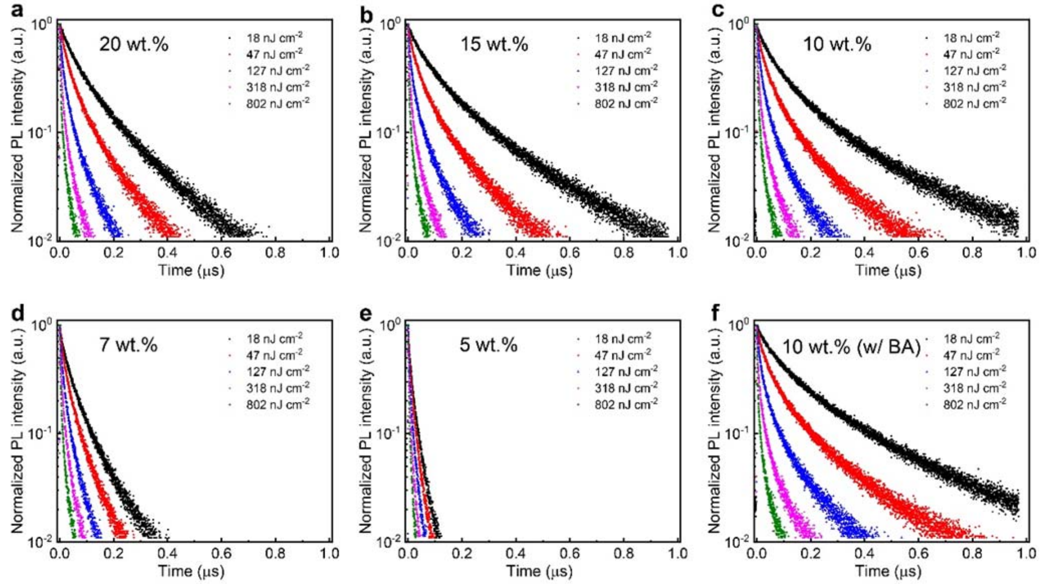

Supplementary Figure 9. Time-resolved PL of perovskite films under various excitation fluence. The films are fabricated with different concentrations of precursor solutions. **a,** 20 wt.%. **b,** 15 wt.%. **c,** 10 wt.%. **d,** 7 wt.% **e,** 5 wt.%. **f,** 10 wt.% with BA treatment.

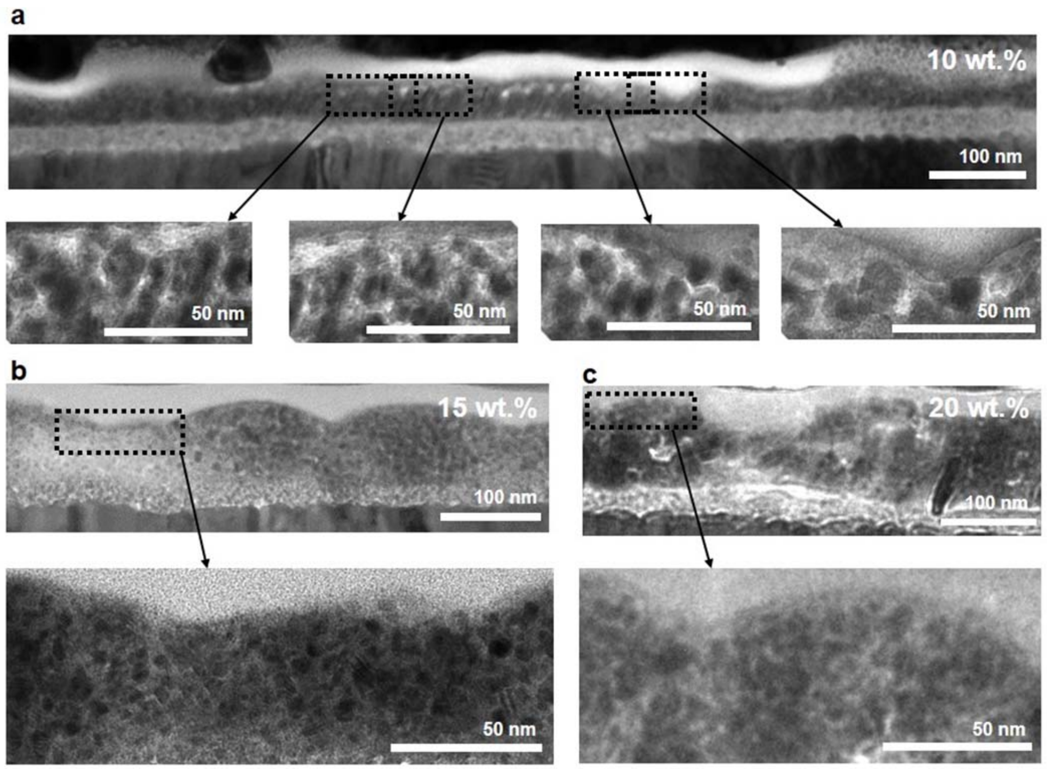

Supplementary Figure 10. Cross-sectional HRTEM images for devices with different concentrations. a, 10 wt.%. **b,** 15 wt.%. **c,** 20 wt.%.

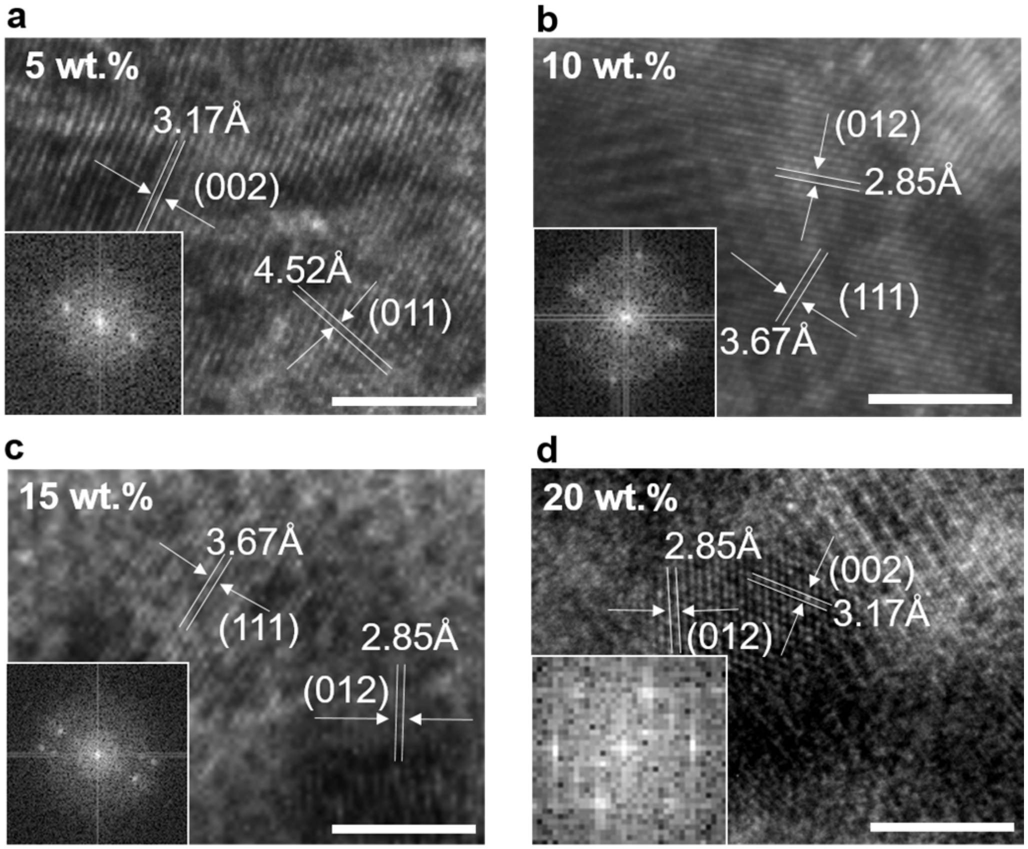

Supplementary Figure 11. Lattice fringe and fast Fourier transformation (FFT) analyses of the perovskite crystals with different concentrations. a, 5 wt.%. **b,** 10 wt.%. **c,** 15 wt.%. **d,** 20 wt.%. These crystals have the cubic structure similar to that of the 3D α-$FAPbI_3$. Scale bar, 5 nm.

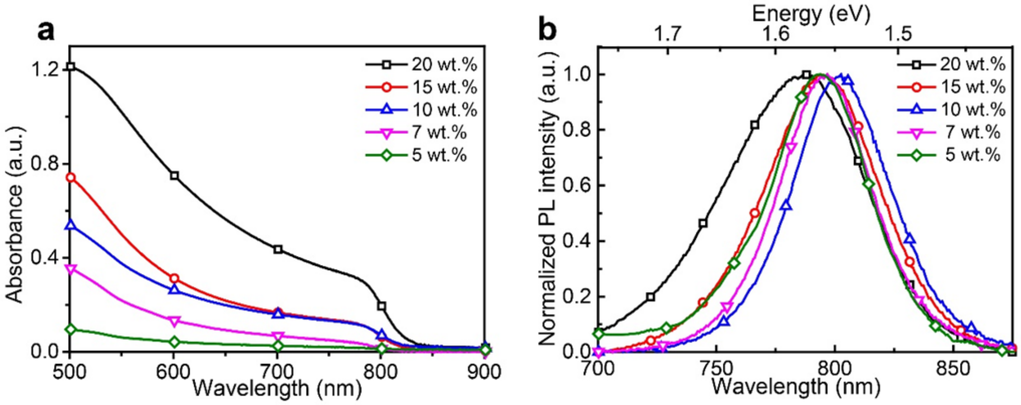

Supplementary Figure 12. Optical properties of $FAPbI_3$ films. The films were fabricated from precursor solutions with different concentrations. **a,** UV-Vis absorption spectra. **b,** PL spectra.

Supplementary Table 1. Stability comparison of our device with NIR OLED and other PeLEDs.

Emission layer	Current density (mA cm^{-2})	Initial radiance (W sr^{-1} m^{-2})	Initial luminance (cd m^{-2})	Initial EQE (%)	Device lifetime (h)	EL peak (nm)	Reference
$FAPbI_3$	100	49.3	-	11.5	$T_{50} \approx 24$	802	This work
$FAPbI_3$ (5-AVA as additive)	100	95	-	18.8	$T_{50} = 20$	803	[1]
$MAPbI_3$	3	~0.5	-	~6.5	$T_{50} > 10$	749	[2]
F8BT:DPPcy (NIR OLED)	10	~0.13	-	~0.45	$T_{50} \approx 60$	740	[3]
$NCPI_6Cl$	10	-	7	2.25	$T_{50} = 5$	688	[4]
$MAPbBr_3$	20	-	~5500	~6	$T_{70} \approx 4$	~542	[5]
$CsPbBr_3$ (MABr capping layer)	166.7	-	7130	~20	$T_{50} = 0.17$	525	[6]
$CsPbBr_3$ (nanocrystal)	~50	-	80	~0.05	$T_{50} = 0.17$	516	[7]

Reference

1. Cao, Y. *et al.* Perovskite light-emitting diodes based on spontaneously formed submicrometre-scale structures. *Nature* **562**, 249 (2018).
2. Zhao, L. *et al. In situ* preparation of metal halide perovskite nanocrystal thin films for improved light-emitting devices. *ACS Nano* **11**, 3957–3964 (2017).
3. Sassi, M. *et al.* Near-infrared roll-off-free electroluminescence from highly stable diketopyrrolopyrrole light emitting diodes. *Sci. Rep.* **6**, 34096 (2016).
4. Zhang, S. *et al.* Efficient red perovskite light-emitting diodes based on solution-processed multiple quantum wells. *Adv. Mater.* **29**, 1606600 (2017).
5. Lee, S. *et al.* Amine-based passivating materials for enhanced optical properties and performance of organic–inorganic perovskites in light-emitting diodes. *J. Phys. Chem. Lett.* **8**, 1784–1792 (2017).
6. Lin, K. *et al.* Perovskite light-emitting diodes with external quantum efficiency exceeding 20 per cent. *Nature* **562**, 245 (2018).
7. Zhang, X. *et al.* Enhancing the brightness of cesium lead halide perovskite nanocrystal based green light-emitting devices through the interface engineering with perfluorinated ionomer. *Nano Lett.* **16**, 1415–1420 (2016).
